# Cognitive dysfunction in diabetic rats is prevented by pyridoxamine treatment. A multidisciplinary investigation

**DOI:** 10.1016/j.molmet.2019.08.003

**Published:** 2019-08-05

**Authors:** Sarah Kassab, Paul Begley, Stephanie J. Church, Sanziana M. Rotariu, Cleo Chevalier-Riffard, Andrew W. Dowsey, Alexander M. Phillips, Leo A.H. Zeef, Ben Grayson, Joanna C. Neill, Garth J.S. Cooper, Richard D. Unwin, Natalie J. Gardiner

**Affiliations:** 1 Faculty of Biology, Medicine and Health, University of Manchester, UK; 2 School of Biological Sciences, University of Auckland, New Zealand; 3 Department of Population Health Sciences and Bristol Veterinary School, Faculty of Health Sciences, University of Bristol, Bristol, BS8 2BN, UK; 4 Department of Electrical Engineering and Electronics, University of Liverpool, UK

**Keywords:** Cognitive decline, Diabetes, Metabolomics, Proteomics, Pyridoxamine, Synaptic plasticity

## Abstract

**Objective:**

The impact of diabetes mellitus on the central nervous system is less widely studied than in the peripheral nervous system, but there is increasing evidence that it elevates the risk of developing cognitive deficits. The aim of this study was to characterize the impact of experimental diabetes on the proteome and metabolome of the hippocampus. We tested the hypothesis that the vitamin B6 isoform pyridoxamine is protective against functional and molecular changes in diabetes.

**Methods:**

We tested recognition memory using the novel object recognition (NOR) test in streptozotocin (STZ)-induced diabetic, age-matched control, and pyridoxamine- or insulin-treated diabetic male Wistar rats. Comprehensive untargeted metabolomic and proteomic analyses, using gas chromatography-mass spectrometry and iTRAQ-enabled protein quantitation respectively, were utilized to characterize the molecular changes in the hippocampus in diabetes.

**Results:**

We demonstrated diabetes-specific, long-term (but not short-term) recognition memory impairment and that this deficit was prevented by insulin or pyridoxamine treatment. Metabolomic analysis showed diabetes-associated changes in 13/82 identified metabolites including polyol pathway intermediates glucose (9.2-fold), fructose (4.9-fold) and sorbitol (5.2-fold). We identified and quantified 4807 hippocampal proteins; 806 were significantly altered in diabetes. Pathway analysis revealed significant alterations in cytoskeletal components associated with synaptic plasticity, glutamatergic signaling, oxidative stress, DNA damage and FXR/RXR activation pathways in the diabetic rat hippocampus.

**Conclusions:**

Our data indicate a protective effect of pyridoxamine against diabetes-induced cognitive deficits, and our comprehensive ‘omics datasets provide insight into the pathogenesis of cognitive dysfunction enabling development of further mechanistic and therapeutic studies.

## Highlights

• Pyridoxamine prevented long-term recognition memory deficits in diabetic rats.
• Hippocampal protein changes are revealed by comprehensive untargeted proteomics.
• Hippocampal metabolomic analysis showed increased polyol pathway intermediates.
• Pyridoxamine altered synaptic plasticity-associated proteins in diabetic rats.

## 1 Introduction

Diabetic neuropathy (DN) is a common secondary microvascular complication of type 1 and type 2 diabetes mellitus [1], [2]. The first symptoms of DN typically manifest in a ‘glove and stocking’ distribution, with distal die-back of sensory axons of the peripheral nervous system (PNS) and associated neuropathic pain, allodynia, paraesthesia, and numbness [3]. Several metabolic perturbations have been linked to the pathogenesis of peripheral DN (including hyperglycemia, dyslipidemia, oxidative stress, altered levels of insulin and neurotrophic factors, polyol-pathway flux, non-enzymatic glycation, and inflammatory stress [4], [5].

The impact of diabetes on the central nervous system (CNS) has been less widely studied than the PNS, but it is increasingly evident that people with diabetes have a higher risk of developing Alzheimer's disease or other CNS disorders related to cognitive decline. Cognitive deficits are observed in several domains including executive function, reduced speed of information processing, attention, and impairments in long-term memory (LTM) [6]. Impairments in memory can range from mild cognitive impairment to more chronic dementia, and are collectively termed “diabetes-associated cognitive decline” (DACD). Understandably this can negatively impact on the quality of life for people with diabetes [6]. Understanding the underlying pathophysiology of these deficits will lead to improved therapeutic strategies to restore cognitive function, and thereby quality of life.

Preclinical studies have described memory and learning impairments, including spatial memory and memory retention deficits in rodents with streptozotocin (STZ)-induced diabetes [7], [8]. A number of changes in the hippocampus, a region associated with recognition memory formation and consolidation, have also been described in STZ-diabetic rodents. Zhang et al. (2008) showed both neurogenesis and neuronal survival were markedly reduced in the hippocampal dentate gyrus of STZ-diabetic rats [9]. Metabolic, structural, and functional modifications that may contribute to neuronal damage include impaired insulin and growth factor signaling and altered signal-transduction pathways [10], [11]; formation of advanced glycation endproducts (AGE) [12]; deposition of neurofibrillary plaques and tangles [5]; and small vessel disease [13]. Thoroughly validated preclinical rodent models are essential to both elucidate pathogenic changes associated with memory dysfunction and test new therapeutic strategies.

Pyridoxamine is one of the three interconvertible members of vitamin B6, along with pyridoxine and pyridoxal. All three forms are biotransformed into physiologically active pyridoxal-5-phosphate which has multiple functions. For example, it is an essential coenzyme/cofactor in processes including metabolism of essential amino acids, glycogen and lipid metabolism, and synthesis of neurotransmitters such as serotonin, dopamine and GABA [14], [15]. Pyridoxamine reduces hyperlipidemia [16] and is a post-Amadori inhibitor (suppressing AGE formation and advanced lipoxidation endproduct formation) [16]. Protective effects of pyridoxamine have previously been described in a number of secondary complications including diabetic cardiovascular disease [17], retinopathy [18], and nephropathy [19] in STZ-diabetic rats.

Here, we describe the presence of long-term recognition memory deficits in STZ-diabetic rats. We test the hypothesis that pyridoxamine is protective against cognitive dysfunction in this context. Then, in order to better understand the underlying molecular pathogenesis of cognitive dysfunction in diabetes, we perform untargeted metabolomic and proteomic analyses of the hippocampus and compare metabolite and protein profiles of age-matched control rats, diabetic rats and diabetic rats treated with pyridoxamine.

## 2 Materials and methods

### 2.1 Animal studies

All reagents were purchased from Sigma–Aldrich (UK) unless otherwise stated. All experiments were conducted using adult male Wistar rats (start weight 300–400 g; Charles River, UK) in accordance with the UK Animals (Scientific Procedures) Act 1986, EU-201063, the ARRIVE guidelines, and ethical approval (University of Manchester).

For each study, rats were randomly allocated (by cage number) into an age-matched control (naïve), untreated-diabetic, or a diabetic-treatment group. Diabetes was induced with an intraperitoneal injection of STZ (55 mg/kg in sterile saline) administered after an overnight fast. Blood glucose levels in tail vein blood were measured 3 days post-STZ using an Accu-chek® Aviva Blood Glucose Meter to confirm hyperglycemia (>15 mmol/L). Rats were housed (controls 3–4; diabetics 2 per cage) at 21 °C in individually ventilated cages (Double-decker cage, Techniplast, UK) under a 12:12hr light:dark cycle (lights on at 7am), with access to standard laboratory chow (Special Diet Services, UK) and water ad libitum. Sizzle nest and burrowing tubes were used to provide environmental enrichment. Rats were checked and weighed regularly, and maintained for 9 (Study 1, 2 and 4) or 12 weeks (Study 3). Treatment protocols: two slow-release insulin pellets (∼4U insulin/day; Linshin, Canada) were implanted subcutaneously (under isoflurane anaesthesia, with 0.001 mg/kg post-operative analgesic buprenorphine) to diabetic rats either at 6 days post-STZ (Study 1) or 6 weeks post-STZ (Study 2). This significantly reduced hyperglycemia but did not restore to control levels (Table 1). Alternatively rats were administered pyridoxamine dihydrochloride (Study 3; 400 mg/L Sigma, UK; Study 4; 1 g/L: Hubei Yuancheng Saichuang Technology Co. Ltd, China) in their drinking water from 1 week post-STZ (Table 1).

**Table 1:** **Indices of Diabetes**. Untreated-diabetic rats were significantly lighter than age-matched control rats by the end of the studies with reduced lean and fat mass, higher blood glucose, free (Study 3) or total (Study 4) cholesterol and triglyceride levels. Pyridoxamine treatment did not alter hyperglycaemia. Pyridoxamine-treated diabetic rats (1 g/L) were significantly lighter than age-matched untreated-diabetic rats, with reduced lean mass by the end of the study (Study 4). However treatment with 400 mg/L pyridoxamine did not impact on end body weight or composition (Study 3). Both concentrations of pyridoxamine reduced diabetes-associated hyperlipidemia. Data are expressed as mean ± standard deviation and analyzed using one-way ANOVA followed by Tukey's post-hoc test. * denotes level of significant difference vs. control rats; # denotes level of significant difference vs. untreated-diabetic rats.

Experimental group *(n numbers*)	Start body weight (g)	End body weight (g)	Terminal blood glucose (mmol/L)	Fat mass (g)	Lean mass (g)	Plasma cholesterol (mg/dl)	Plasma triglyceride (mg/dl)
**Study 1**							
Age-matched control *(n = 10)*	336 ± 15	457 ± 28	5.43				
Untreated-diabetic *(n = 10)*	348 ± 7	403 ± 26****	28****				
Insulin-diabetic (at 6 days post-STZ) *(n = 7)*	354 ± 25	540 ± 72 ####	17.9**** ###				
**Study 2**							
Age-matched control *(n = 10)*	383 ± 16	548 ± 44	8.5				
Untreated-diabetic *(n = 9)*	367 ± 22	379 ± 42****	54.4****				
Insulin-diabetic (at 6 weeks post-STZ) *(n = 9)*	358 ± 15*	411 ± 40****	33.5**** ###				
**Study 3 (400 mg/L)**							
12 week duration Age-matched control *(n = 12)*	333 ± 14	557 ± 33	10.13	69 ± 25	431 ± 29	30 ± 5	175 ± 73
*Body weight at 9 weeks post-STZ (g)*		*533 ± 35*					
Untreated-diabetic *(n = 10)*	338 ± 14	392 ± 41****	30.4****	24 ± 7****	304 ± 41****	78 ± 32****	607 ± 217****
*Body weight at 9 weeks post-STZ (g)*		422 ± 93**					
Pyridoxamine-diabetic (400 mg/L) *(n = 9)*	325 ± 14	384 ± 37****	31.98****	23 ± 5****	304 ± 35****	47 ± 20#	289 ± 223##
*Body weight at 9 weeks post-STZ (g)*		399 ± 72***					
**Study 4 (1** **g/L)**							
9 week duration							
Age-matched control *(n = 12)*	365 ± 34	517 ± 66	7.1	64 ± 22	406 ± 44	208 ± 52	189 ± 137
Untreated-diabetic *(n = 10)*	389 ± 18	408 ± 44****	35.88****	22 ± 2****	311 ± 46****	487 ± 245**	674 ± 277****
Pyridoxamine-diabetic (1 g/L) *(n = 14)*	371 ± 28	354 ± 23**** #	33.3****	18 ± 5****	260 ± 31**** ####	291 ± 176#	358 ± 242##

* denotes level of significant difference vs. control rats; ** (p < 0.01), ** (p < 0.001), **** (p < 0.0001), # denotes level of significant difference vs. untreated-diabetic rats; ## (p < 0.01), ### (p < 0.001), #### (p < 0.0001).

### 2.2 Novel object recognition test

NOR was conducted at 8 weeks post-STZ. Rats were acclimatized to an empty square open-field testing arena for 10 min over 2 consecutive days (between 9am and 1pm). The next day, for the acquisition phase, each rat was placed in the test arena now containing two identical objects (weighted bottles or metal cans) positioned 6 cm in from diagonal corners. The rat was filmed for 3 min then returned to its home cage for an inter-trial interval (ITI) of either 3 min or 1 h. Both the arena and objects were cleaned after each trial to remove olfactory cues. Following the ITI, rats were returned to the arena which now contained a triplicate of the familiar object from the acquisition phase and a novel object (retention trial) and were filmed for 3 min. Behavior was analyzed from coded video recordings by trained experimenters blinded to the treatment groups. Exploration time of each object (time spent sniffing or touching the object but not standing, sitting on, or leaning against the object) was measured. The discrimination index (DI= (T_NO_ – T_FO_)/(T_NO_ + T_FO_)), was calculated - where T_NO_ is the exploration time of the novel object and T_FO_ is the exploration time of the familiar object^(24)^. Locomotor activity was measured as the number of floor gridlines crossed by the base of the rat's tail.

### 2.3 Tissue harvest

At the end of the study, core blood glucose was measured from terminally-anaesthetized rats (isoflurane) that were culled by decapitation. A sample of core blood was also collected into lithium heparin tubes (Greiner), centrifuged at 2500/3000rpm for 20 min at 4 °C and plasma/serum supernatant stored at −80 °C. The composition of the cadavers (Studies 3 and 4) was immediately assessed using an EchoMRI system (Echo Medical Systems) to determine proportions of fat and lean body mass. Whole brains or hippocampi were dissected, snap-frozen, and stored at −80 °C. Free or total cholesterol and triglyceride levels were determined using Cholesterol Fluorometric Assay and Triglyceride Colorimetric Assay kits (Cayman Chemical, USA).

### 2.4 Statistical analysis

Exclusion criteria for in vivo experiments are shown in Table 2. Data are presented as individual animal data, and as group mean (±standard deviation) or median (±interquartile range) as appropriate for datasets. GraphPad Prism 7.0 was used for statistical analysis: paired t-tests/Wilcoxon matched-pairs test, one-way ANOVA/Kruskal–Wallis test, two-way ANOVA/Friedman test with appropriate post hoc tests as stated in the text (^#/∗^p < 0.5, ^##/∗∗^p < 0.01, ^###/∗∗∗^p < 0.001, ^####/∗∗∗∗^p < 0.001).

**Table 2:** Exclusion Criteria for in vivo studies and analysis.

Reversion to normoglycaemia (<16 mmol/L)	Rat culled/data excluded from all analysis
Evident morbidity and/or rapid weight loss not controlled by implantation of half-pellet of slow-release insulin (∼1U insulin/day; Linshin, Canada; under isoflurane anaesthesia, with 0.001 mg/kg post-operative analgesic buprenorphine) to maintain welfare. This dose does not correct hyperglycaemia.	Rat culled/data excluded from all analysis
Failed to explore one or both objects in NOR acquisition or retention trial	All NOR data for that rat excluded from analysis
One of objects knocked down in acquisition or retention phase	All NOR data for that rat excluded from analysis

### 2.5 Metabolomic analysis of the hippocampus

50±5 mg hippocampal tissue (Study 3) were extracted in 800 μl of cold 50:50 (v/v) choloroform:methanol containing a mixture of isotopically labeled internal standards in methanol as previously described [20]. Two extraction blanks (no sample) were also included. LC-MS grade water (400 μl) was added to initiate phase separation, samples were vortexed and centrifuged (2400×*g*, 15 min). 200 μl of the polar (methanol:water) phase from each sample (individual animals) was collected and quality control standards also prepared by pooling 200 μl from each sample. Samples and standards were dried using a Savant Speedvac centrifugal concentrator (ThermoFisher Scientific, UK) and stored at 4 °C.

### 2.6 GC–MS derivatisation and run

Polar phase samples, blanks and QC standards were chemically derivatized as described previously^(20)^ to methoxime/trimethylsilyl derivatives, then analyzed by GS-MS using a Agilent/J&W DB17-MS column (30 m × 0.25 mm × 0.25 μm), a 3 m × 0.25 mm retention gap and helium carrier with a constant flow rate of 1.4 ml/min and a Pegasus high-throughput time-of-flight mass spectrometer (LECO; UK).

### 2.7 GC–MS data processing and analysis

The “Reference Compare” method was used to prepare the mass spectral data for analysis using the ChromaTOF 4.5 software (LECO; UK). Putative metabolites were identified from the NIST Mass Spectral Reference Library (NIST08/2008; NIST, Gaithersburg, USA) and an in-house library. GraphPad Prism 7.0 was used for statistical analysis.

### 2.8 Proteomic analysis of hippocampus

#### 2.8.1 iTRAQ labeling peptide mixture preparation

Hippocampi from control, untreated-diabetic and pyridoxamine-treated diabetic rats (study 4) were washed with PBS and lysed in 400 μl 1M triethylammonium bicarbonate (TEAB) and 0.1% w/v sodium dodecyl sulfate (SDS) using a TissueLyserII (3 min at 25 Hz). Samples were centrifuged (10 min at 4 °C, 12000rpm) and supernatant collected. Protein concentrations were determined, 100 μg of protein from each sample was aliquoted and volumes equalized to 30 μl with lysis buffer. Cysteine reduction, alkylation, and digestion of proteins were conducted [21]. Resulting tryptic digests were dried, resuspended in 20 μl 1M TEAB and peptides labeled using 8-plex iTRAQ reagent according to the manufacturer's instructions (AB Sciex). Samples were dried for 15min to remove ethanol, then 100 μl of loading buffer (2% acetonitrile, 0.1% ammonium bicarbonate, pH > 10) was added to each sample. Labeled samples were pooled, made up to 1.8 ml with loading buffer and stored at −20 °C.

#### 2.8.2 High-performance liquid chromatography

Samples were thawed, centrifuged, and peptides fractionated off-line using high-pH reversed-phase chromatography on a 3 μm Extend-C18 column (4.6 × 100mm; Agilent, UK) on an Agilent 1200 series LC system at 45 °C using a 30 min gradient from 3% to 40% acetonitrile in 0.1% ammonium hydroxide at 0.75 ml/min 30-s fractions were collected, dried, and stored at −20 °C until analysis.

For analysis by low-pH reversed-phase LC-tandem MS analysis, dried fractions were resuspended in 10 μL 3% (v/v) acetonitrile and 0.1% (v/v) trifluoroacetic acid, with 1 μL analyzed by low-pH reversed-phase chromatography using a nanoACQUITY UHPLC system (Waters) online to a Triple-Tof 6600 mass spectrometer (AB Sciex) as previously described [21].

#### 2.8.3 Proteomic analysis

Raw data files were analyzed using ProteinPilot 5.0 with default search settings against a rat-specific Uniprot database (Uniprot_Rat_Nov17; 29,997 proteins) as described previously [22]. Identified peptides were then coalesced to protein-level quantifications and statistical testing for differential expression performed using v1.0.0 of the in-house developed software ‘BayesProt’ (https://github.com/biospi/bayesprot/releases/tag/v1.0.0). An earlier version of this technique was presented in Freeman et al. [22], which combined Protein-Pilot (AB SCIEX) sample normalization (‘bias correction’) with a Bayesian linear mixed-effects model implemented with the MCMCglmm R Package [Hadfield, Jarrod D. “MCMC methods for multi-response generalized linear mixed models: the MCMCglmm R package.” Journal of Statistical Software 33.2 (2010): 1–22.].

Since iTRAQ measurements from Time-of-Flight instruments are recorded as discrete ion counts, and technical/biological variation are assumed log-normal, we adopted a Generalized Linear Mixed Model (GLMM) with Poisson likelihood and log-link, where each protein was modeled separately using peptide measurements unique to that protein. The sample normalization factors represent the mass spectrometer's exposure to each sample, and hence were included as a fixed offset within the model. The current version of BayesProt additionally (i) enables estimation of both biological and digestion variance through the incorporation of multiple digests for a single sample (i.e. the six reference pool digests), (ii) negates the need for Protein-Pilot normalization by implementing a two-stage GLMM and (iii) provides a simplified Markov Chain Monte Carlo (MCMC) mixing criterion for both stages.

In both stages: (a) for each peptide a separate random digest effect is fitted, which has the effect of weighting each peptide's contribution to the protein-level quantification by its reproducibility across digests; (b) the set of measurement channels within each iTRAQ spectrum are each assigned (i) a baseline fixed effect to account for varying selection/ionisation/fragmentation efficiencies across spectra, and (ii) an independent log-normal residual variance to account for over-dispersion due to background contamination and incorrectly identified spectra. In stage one, we also model the interaction between LC-MS/MS run and iTRAQ channel as a fixed effect i.e. within each run, we infer the protein-level log ratio between iTRAQ channel 113 and channels 114, 115, 116, 117, 118, 119, and 121. For each channel relative to 113, the result is a set of posterior probability distributions, one for each protein in the study; these are combined to derive a posterior distribution for the median log ratio for each channel relative to 113, which is taken as the inferred sample normalization factors.

In stage two, rather than using point estimates of the normalization factors as fixed sample offsets, a set of sample fixed effects are fitted which have prior distributions set to the means and variances of the inferred median log ratio distributions. In addition, in stage two, we specify the full experimental design: (a) protein-level differential expression fold change between cases and controls is fitted as a condition fixed effect (with control as baseline); (b) a random effect is fitted across samples. For the comparisons untreated-diabetic vs. control and diabetic-pyridoxamine vs. untreated-diabetic, using the inferred posterior distribution of the condition fixed effect, for each protein we based our one-sided significance test on the posterior probability that the mean fold change was at least 5% above or below control or diabetic untreated expression and defined a significant difference in protein expression using a global false discovery rate (FDR) threshold of 10% *i*.*e*. the largest set of proteins with an average FDR <10% were deemed significant.

Residual variances were assigned inverse-Gamma priors, while random effects were assigned parameter-expanded Cauchy priors. The model was tested with different prior scale factors to establish that the priors were not informative to the outcome. In stages one and two, the model was run with 10 and 100 MCMC chains per protein, respectively, each chain consisting of 10,000 samples preceded by 3,000 burn-in samples. Mixing was assessed using Warnes & Raftery's MCGibbsit run-length diagnostic, combining the estimate error-bounding approach of Raftery and Lewis with the between chain variance verses within chain variance approach of Gelman and Rubin (https://cran.r-project.org/web/packages/mcgibbsit/index.html).

Following Bayesian analysis, protein lists were analyzed using Ingenuity Pathway Analysis (IPA; QIAGEN [www.qiagen.com/ingenuity]). To identify enrichment of pathways in our list of changes (global FDR 0.1) compared with the whole user input data set. STRING (Version 10.5) [23] was used to analyze pathway enrichments and physical interactions between the significantly changed proteins.

## 3 Results

### 3.1 Diabetic rats showed deficits in long–term (but not short term) recognition memory which is ameliorated by treatment with insulin

**Figure 1:**
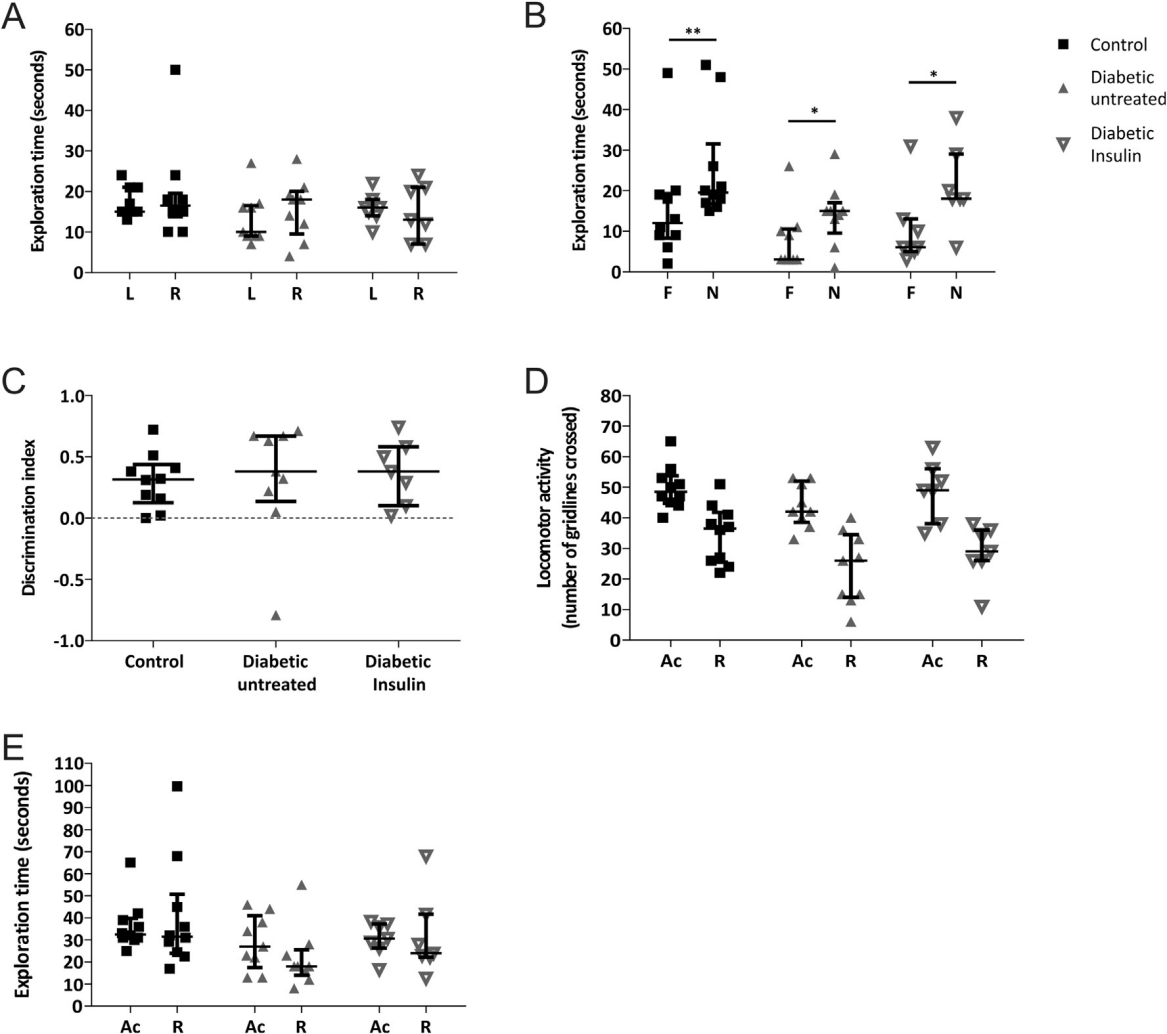
**Novel object recognition test analysis revealed no evidence of deficits in short-term recognition memory (3 min ITI) in STZ-diabetic rats (Study 1). A)** All experimental groups showed no evidence of object preference (L: left and R: right) during the 3 min acquisition (Ac) phase. **B)** Control (*n* = *10*; **p < 0.01), diabetic-untreated rats (*n* = *9*; *p < 0.05) and insulin-treated diabetic rats (*n* = *7*; **p < 0.01) explored the novel object (N) for a significantly longer time than the familiar object (F) in the retention (R) phase. **C)** The Discrimination Index reveals all experimental groups preferentially explored the novel object significantly more than the familiar object. All experimental groups showed similar levels of **D)** locomotor activity and **E)** total exploration time in the Ac and R phases. All data are represented as median ± interquartile range. Data are analyzed using Wilcoxon matched-pairs test between L and R **(A)** or F and N **(B)** for each experimental group, Kruskal–Wallis test followed by Dunn's post-hoc test **(C)** or Freidman test followed by Dunn's post hoc test **(D&E)**.

Here we utilized the ethologically relevant Novel Object Recognition (NOR) test [24] to verify that STZ-diabetic rats develop diabetes-associated cognitive deficits and investigate the effect of insulin treatment. An NOR test of age-matched control, untreated-diabetic and insulin-treated diabetic rats (Study 1) was conducted 8 weeks post-STZ. Indices of diabetes are shown in Table 1. All experimental groups explored both objects for similar amounts of time during the acquisition phase (Figure 1A). Following a 3min ITI, all experimental groups showed a significant preference for exploring the novel object over the familiar one (Figure 1B,C). All experimental groups displayed similar levels of locomotor activity (Figure 1D), no evidence of lethargy and comparable total exploration times (Figure 1E) in both phases.

This result indicates that: 1) systemic administration of STZ has not itself caused short-term memory (STM)-deficits (important since intracerebroventricular administration of STZ has been used as a preclinical model of sporadic Alzheimer's disease with associated cognitive deficits [25]); 2) diabetic rats can be used in the NOR testing paradigm i.e. they have the physical and cognitive abilities to identify and distinguish between familiar and novel objects; and 3) diabetic rats have no STM deficits in object recognition.

**Figure 2:**
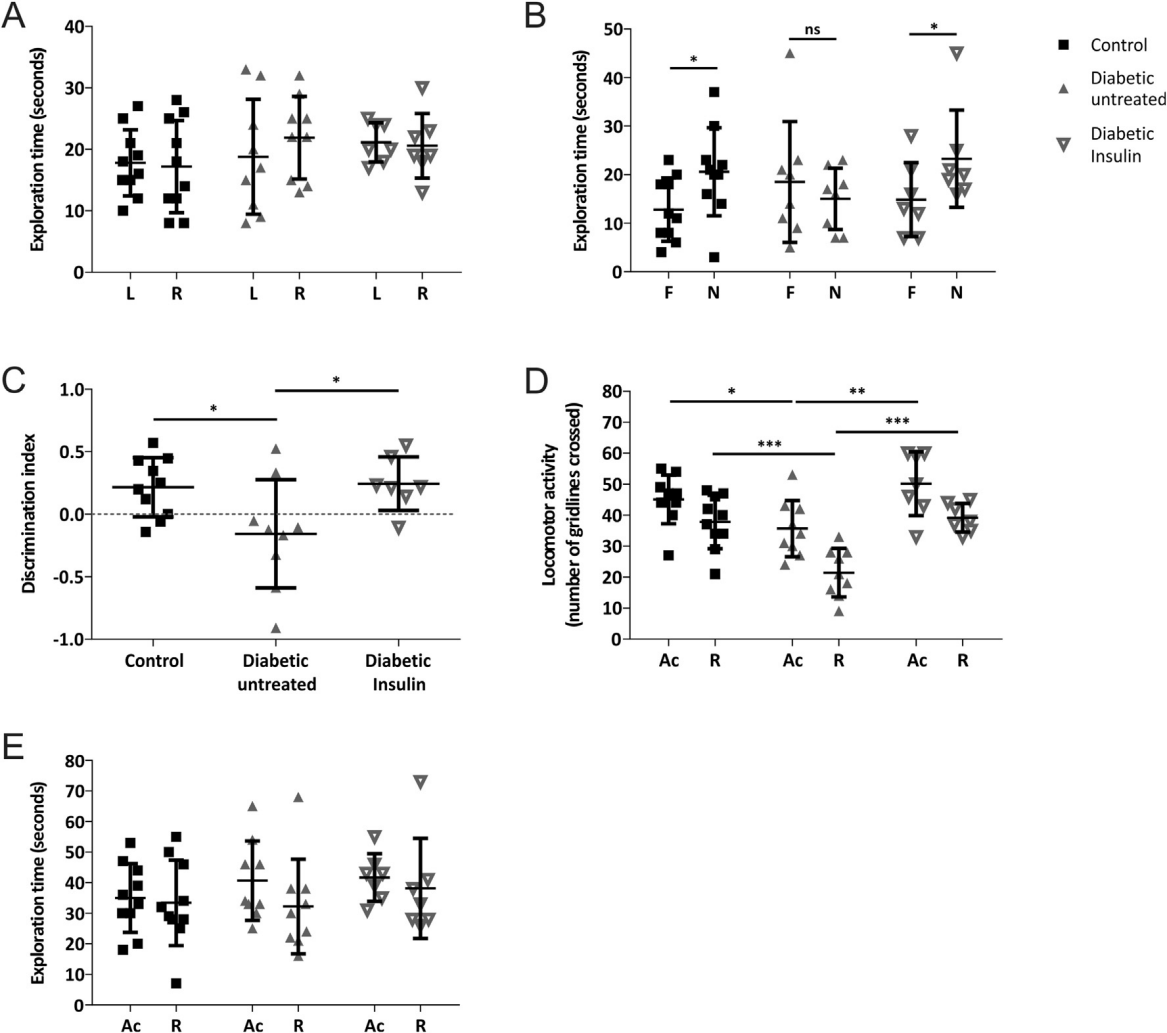
**Novel object recognition test analysis showed a deficit in longer-term recognition memory in STZ-diabetic rats (ITI of 1 h) that was prevented by insulin treatment (Study 2). A)** All experimental groups showed no evidence of object preference (L:left and R:right) during the Acquisition (Ac) phase **B)** Control (*n* = *10*) and insulin-treated diabetic rats (*n* = *7*) explored the novel (N) object significantly more than the familiar (F) object (*p < 0.05), whereas the untreated-diabetic rats (*n* = *9*) showed no evidence of object preference during the retention (R) phase. **C)** The discrimination index reveals control and insulin-treated diabetic rats preferentially explored the novel object significantly more than the diabetic untreated rats (*p < 0.05). **D)** In both phases the diabetic untreated rats displayed significantly reduced locomotor activity compared to control (*p < 0.05; ***p < 0.001) and insulin-treated diabetic rats (**p < 0.01; ***p < 0.001). **E)** Total exploration time did not differ significantly between experimental groups. All data are represented as mean ± standard deviation. Data are analyzed by paired t-test between L and R **(A)** or F and N **(B)**, one-way ANOVA followed by Tukey's post-hoc test **(C)**, or two-way ANOVA followed by Tukey's post-hoc test **(D&E)**.

Thereafter, we conducted the NOR test in a new group of rats at 8 weeks (Study 2: control, untreated-diabetic and diabetic rats treated with insulin from 6 weeks post-STZ), using a 1 h ITI to assess longer-term recognition memory. As in the previous study, rats showed no evidence of object preference in the acquisition phase (Figure 2A). Following the ITI control rats exhibited a preference for the novel object (Figure 2B,C; p < 0.05). In contrast untreated-diabetic rats explored both objects equally, showing no evidence of object recognition (Figure 2B&C; p > 0.05). This recognition memory deficit was not evident in the insulin-treated diabetic rats, which showed a preference for the novel object (Figure 2B,C Discrimination Indices: control: 0.22 ± 0.24; diabetic untreated: −0.16 ± 0.4; diabetic insulin-treated: 0.24 ± 0.21). It is important to note that while the untreated-diabetic rats displayed less locomotor activity compared to age-matched control and insulin-treated diabetic rats in both phases of this particular study (Figure 2D), the total object exploration times in both phases were comparable between all groups of rats (Figure 2E). This provides evidence that longer-term recognition memory deficits occur in diabetic rats and that these deficits are diabetes-associated, can be ameliorated by insulin treatment and are therefore not an indirect effect of STZ.

### 3.2 Pyridoxamine treatment prevented deficits in recognition memory

We assessed the therapeutic potential of pyridoxamine to prevent recognition memory deficits at 8 weeks post-STZ in two independent trials, treating drinking water with either 400 mg or 1 g pyridoxamine/L, from 1 week post-STZ. Neither concentration of pyridoxamine altered diabetes-evoked hyperglycemia, but both reduced hyperlipidemia. Treatment with 400 mg/L pyridoxamine did not affect the body weight of diabetic rats, but 1 g/L caused a reduction in lean mass and the treated-diabetic rats weighed less than untreated-diabetic rats at 9 weeks (Table 1).

**Figure 3:**
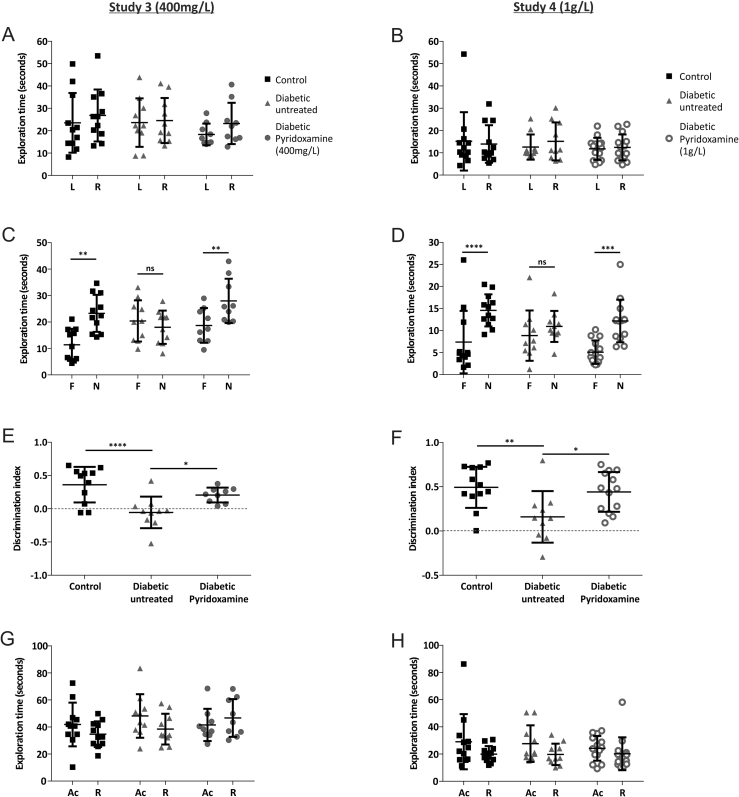
**Pyridoxamine treatment improved recognition memory in diabetic rats.** All experimental groups showed no evidence of object preference in the Acquisition phase of study 3 **(A)** and study 4**(B)**. Control (*n* = *11* study 3 and *n* = *12* study 4) and pyridoxamine-treated diabetic rats (*n* = *9* study 3; n = 14 study 4) explored the novel object significantly more than the familiar one (**C&D**: **p < 0.01 study 3; ****p < 0.0001, ***p < 0.001 study 4), whereas the untreated-diabetic rats (*n* = *10* study 3 and n = 10 study 4) showed no evidence of object preference in the retention phase. Discrimination indices are shown (**E&F**: study 3 ***p < 0.001; *p < 0.05; study 4 **p < 0.001; *p < 0.05). All experimental groups displayed significantly similar levels of exploratory activity in both the acquisition and retention phases (p > 0.05) for both studies (**G&H**). All data are represented as mean ± standard deviation. Acquisition phase (**A&B)** and retention phase (**C&D**) data analyzed using paired t-test between L and R (**A&B**) or F and N (**C&D**) for each experimental group. Discrimination index (**E, F**) data analyzed using one-way ANOVA followed by Tukey's post-hoc test. Total exploration time (**G&H**) data analyzed using two-way ANOVA followed by Tukey's post-hoc test.

All rats explored objects equally during the acquisition phase (Figure 3A,B) and, following the 1 h ITI, control rats showed a significant preference for the novel object in both studies (Figure 3C–F). Untreated-diabetic rats had no apparent preference for either object (Figure 3E–F, p > 0.05), confirming the diabetes-associated LTM deficit previously observed (Figure 2).

Interestingly, we found that diabetic rats treated with pyridoxamine explored the novel object at a level similar to the control rats and significantly different to the untreated-diabetic rats (Figure 3E: 400 mg/L, Discrimination Index: Control 0.361 ± 0.27; Untreated-Diabetic −0.055 ± 0.24; Diabetic-Pyridoxamine 0.206 ± 0.11; Figure 3F: 1 g/L, Discrimination Index: Control 0.493 ± 0.23; Untreated-Diabetic 0.159 ± 0.29; Diabetic-Pyridoxamine 0.441 ± 0.22) indicating protection against diabetes-induced recognition memory deficits. In both studies, there was similar cumulative object exploration during both phases (Figure 3G,H).

Together these data indicate that diabetic rats exhibit DACD at 8 weeks post-STZ, in the form of impaired longer-term recognition memory processes, and that this deficit can be ameliorated by pyridoxamine treatment. In order to explore the pathogenic changes which occur within the hippocampus in diabetes and determine potential mechanisms for the therapeutic role of pyridoxamine, we performed unbiased comprehensive metabolomics and proteomic analysis of the hippocampus.

### 3.3 Experimental diabetes was associated with metabolic dysfunction in the hippocampus

**Figure 4:**
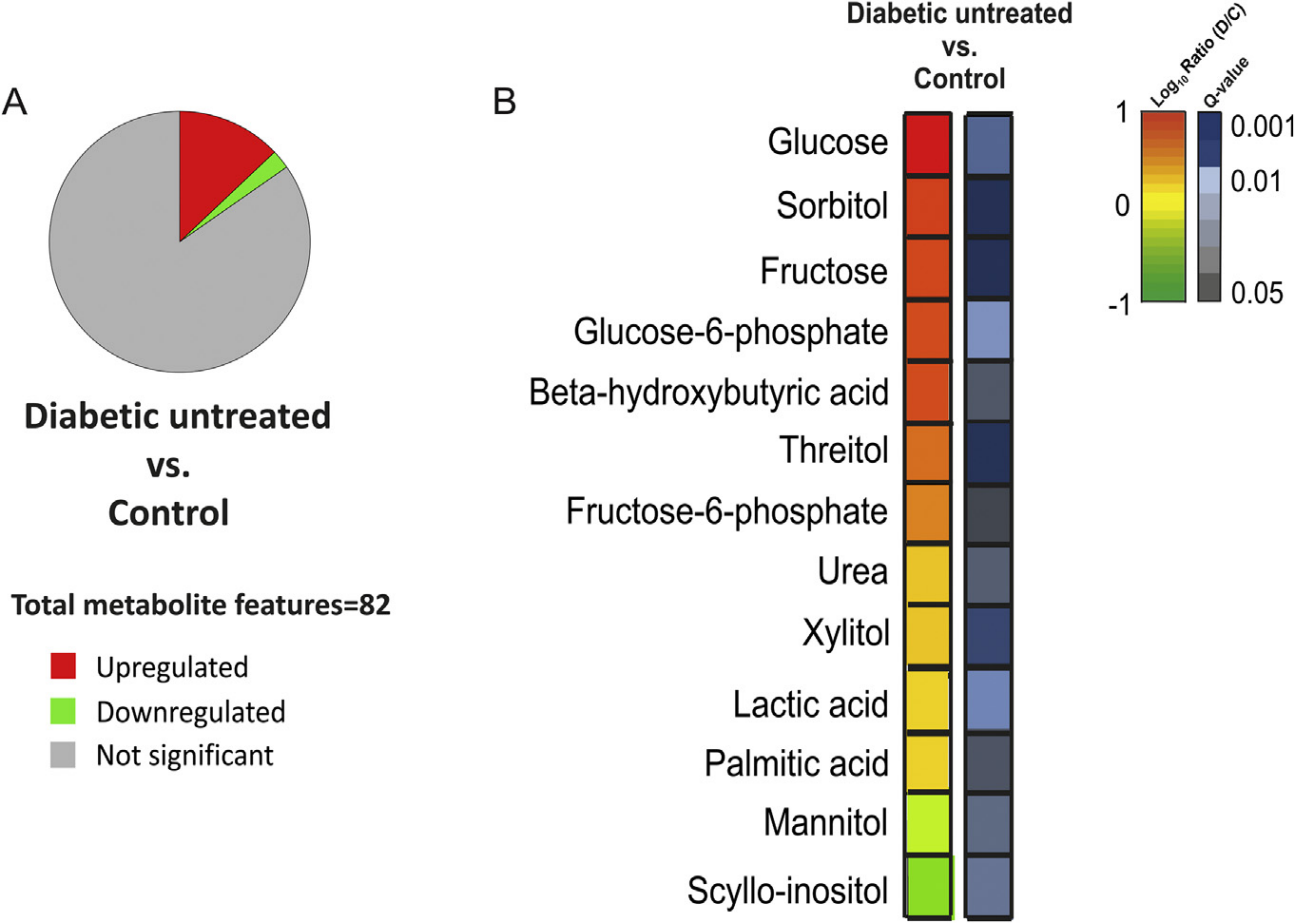
**Metabolite changes in the hippocampus of rats 12 weeks post-STZ revealed a pattern of metabolic dysfunction characteristic of polyol pathway activation**. (**A**) The percentage of identified and quantified metabolites that were up-regulated in the hippocampus in diabetes are shown in red (and include glucose, fructose and sorbitol), those down-regulated are shown in green (including scyllo-inositol) and in grey those not significantly changed (FDR>5%) between diabetic (*n* = *7*) and control (*n* = *6*) rats (Study 3). (**B**) The significantly altered polar metabolites are shown as log10 ratio of fold change (diabetes/control) and Kruskal–Wallis test for each metabolite (with correction for multiple comparisons; FDR of 5%, q value (D/C) according to the key).

Metabolomic analysis of hippocampus of age-matched control (*n* = *6)* and untreated-diabetic rats (*n* = *7*; 12 weeks post-STZ) identified and quantified 82 metabolite features (Figure 4A, all data accessible at https://doi.org/10.17632/n6z95235zx.1). Of these metabolites, 13 were significantly altered - with 11 (13.4%) up-regulated and 2 (2.4%) down-regulated in diabetes (Figure 4A). Similar to our previous ‘omics study of the PNS in DN [22], metabolites involved in the polyol pathway – glucose (9.2-fold, q = 0.0059), fructose (4.9-fold, q = 0.0019) and sorbitol 5.2-fold, q = 0.0015) - showed the greatest increase (Figure 4B) with a 0.27-fold decrease (q = 0.0252) observed in scyllo-inositol levels. Urea (1.6-fold, q = 0.033) and members of the glycolysis pathway, glucose-6-phosphate (4.6-fold, q = 0.0159) and frucose-6-phosphate (2.8-fold, q = 0.0419) were significantly increased in the hippocampus (Figure 4B) indicating that parallels exist between PNS and CNS metabolic dysfunction in diabetes.

Treatment with pyridoxamine (*n* = *4*), significantly altered four (4.9%) of the identified metabolites compared to untreated-diabetic rats with two being up-regulated (2.6%; pyrophosphate and 2-pyrrolidinone) and two down-regulated (2.6%; phenylalanine and tyrosine) in pyridoxamine-treated rats (https://doi.org/10.17632/n6z95235zx.1).

### 3.4 Proteomic analysis revealed dysregulation in metabolic and synaptic pathways in diabetic-rat hippocampus and alterations in cytoskeletal associated proteins by pyridoxamine

**Figure 5:**
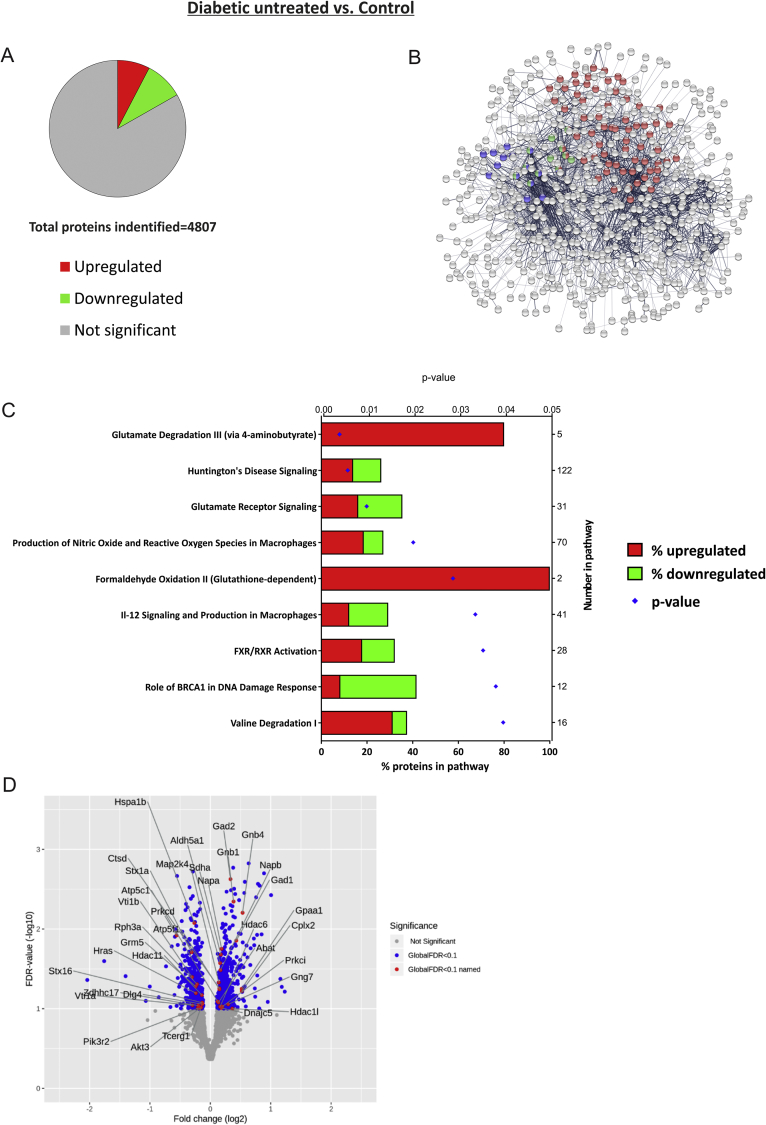
**Proteomic pathway analysis revealed dysregulation in glutamatergic, oxidative and nitrative stress and inflammatory pathways in the diabetic rat hippocampus. A)** Of the 4807 identified and quantified proteins 17% were significantly altered (FDR<10%) in the hippocampus of diabetic (*n* = *6*) vs. control (*n* = *4*) rats (Study 4; up-regulated (red) or down-regulated (green)). **B)** STRING Network analysis highlights interaction networks for significantly altered proteins and the proteins in most significant KEGG pathways are represented as colored nodes (Red: ‘*Metabolic pathways*’, Blue: ‘*Glutamatergic synapse*’ and Green: ‘*GABAergic synapse*’). **C)** Ingenuity Pathway Analysis reveals 9 significantly overrepresented pathways organized by P value (shown on top x-axis); the bars show percentage of proteins in each pathway (bottom x-axis) that are up-regulated (red) or down-regulated (green), total number of proteins within each named pathway is shown on the right-hand Y axis. **D)** Volcano plot showing the distribution of total protein expression arranged by log_2_-fold change and FDR. Significantly changed proteins (FDR<10%) are colored blue and the proteins from the top 2 canonical pathways in the IPA analysis (**C**: “*Glutamate degradation III*” and “*Huntington's Disease signaling*”) are labeled in red (as their gene names).

Comprehensive iTRAQ proteomics of hippocampal protein extracts from control (*n* = *4*), untreated-diabetic (*n* = *6*), and pyridoxamine-treated diabetic rats (*n* = *6*) was used to study differential protein expression. We identified and quantified a total of 4807 proteins in the samples (all data accessible at: https://doi.org/10.17632/72n3ds7hhg.1) and of the 806 (17% total) proteins that were significantly changed in diabetes - 368 (45.7%) were up-regulated and 438 (54.3%) were down-regulated compared to controls (Figure 5A).

The top 3 KEGG pathways ‘*metabolic pathways’* (FDR 0.000038), ‘*glutamatergic synapse’* (FDR 0.000492) and ‘*GABAergic synapse’* (FDR 0.0022) are shown in STRING network analysis (Figure 5B). Ingenuity Pathway Analysis (IPA) revealed the overrepresented canonical pathways of significantly altered hippocampal proteins from diabetic rats compared to controls. These include pathways related to oxidative stress, DNA damage and FXR/RXR activation, with the most significant alterations occurring in glutamate receptor signaling and degradation related pathways (“*Glutamate degradation III* via *4-aminobutyrate*” and ‘*Huntington's disease signaling’* Figure 5C; p < 0.01). All differentially expressed proteins within these two most significantly altered pathways are labeled on the Volcano plot (Figure 5D).

**Figure 6:**
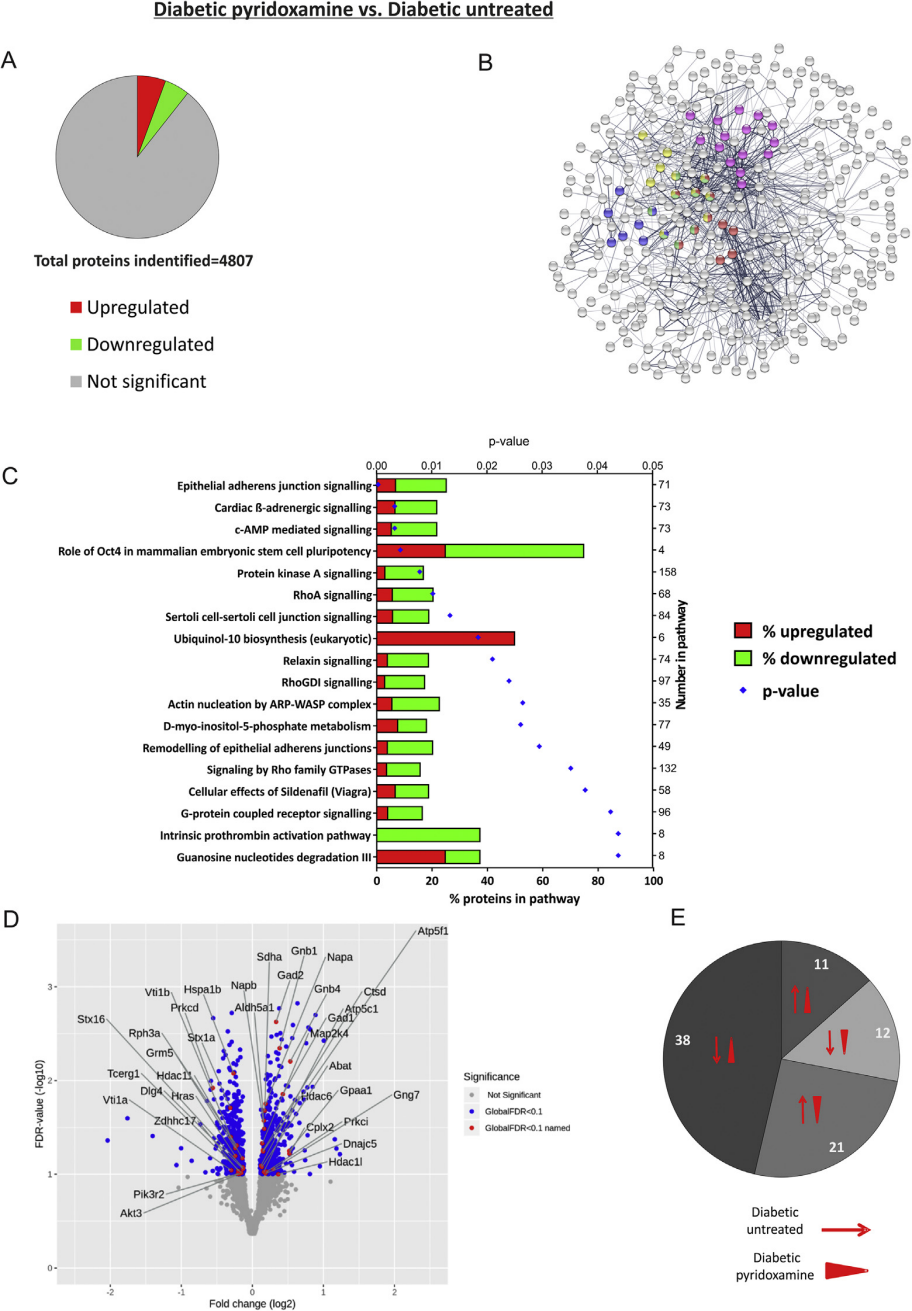
**Proteomic pathway analysis reveals alterations in cytoskeletal-associated proteins and signal transduction pathways in the hippocampus of pyridoxamine-treated diabetic rats. A)** 511 of the identified proteins were significantly up-regulated (red) and down-regulated (green) in the hippocampus of pyridoxamine-treated (*n* = *6*) compared to untreated-diabetic rats (*n* = *6*) rats (Study 3). **B)** STRING Network analysis highlights networks and association for significantly altered proteins and proteins in most significant KEGG pathways are represented as colored nodes (Red: “*Morphine addiction*”, Blue: “*Synaptic vesicle cycle*”, Green: “*Retrograde endocannabinoid signaling*’, Yellow: “*Glutamatergic synapse*”, Pink: “*Regulation of actin cytoskeleton*”). **C)** Ingenuity Pathway Analysis highlights 18 significantly changed pathways organized by P value (shown on top x-axis); the bars show percentage of proteins in pathway (bottom x-axis) that are up-regulated (red) and down-regulated (green), total number of proteins within each named pathway is shown on right-hand Y axis. **D)** Volcano plot shows distribution of total protein expression by log_2_-fold change and FDR. Significantly changed proteins (FDR<10%) are colored blue, and proteins from the top canonical pathway (**C**: “*Epithelial adherens junction signaling*”) are labeled in red (as their gene names). **E)** Pie chart shows the expression profiles of the 82 proteins that were significantly changed in both control vs diabetic and diabetic-untreated vs. diabetic-pyridoxamine datasets. Those up or down-regulated in diabetes compared to controls are shown as thin red arrow and those up- or down-regulated in pyridoxamine-treated diabetic rats compared to untreated-diabetic rats are shown as red arrowheads (e.g. 21 proteins were up-regulated in diabetes and down-regulated in pyridoxamine treated rats).

Analysis of the 511 proteins that were significantly altered in pyridoxamine-treated compared to untreated-diabetic rats revealed that 272 (53.2%) were up-regulated and 239 (46.8%) down-regulated (Figure 6A). The top 5 KEGG pathways ‘*morphine addiction’* (FDR 0.000709), ‘*synaptic vesicle cycle’* (FDR 0.00266), ‘*retrograde endocannabinoid signaling’* (FDR 0.00266), ‘*glutamatergic synapse*’ (FDR 0.00266) and ‘*actin cytoskeleton’* (FDR 0.00266) are shown in STRING network analysis (Figure 6B). IPA highlighted significant overrepresentation of pathways associated with signal transduction (including PKA, RhoA and cAMP pathways) and the most significant “*Epithelial adherens junction signaling*” a pathway associated with cytoskeletal proteins (Figure 6C). Significantly changed proteins from this pathway are highlighted on the Volcano plot (Figure 6D).

Manual inspection of datasets revealed 82 proteins which were significantly altered in both datasets (i.e. untreated-diabetic vs. control and diabetic-pyridoxamine vs. untreated-diabetic, https://doi.org/10.17632/72n3ds7hhg.1). The greatest number of these (38; 46%) were down-regulated in diabetes compared to control, and up-regulated in pyridoxamine-treated compared to untreated-diabetic rats. 21 proteins were up-regulated in diabetes and down-regulated in pyridoxamine-treated rats, and the remaining 23 were either both significantly up- or down-regulated (Figure 6E). The dynamic expression of these proteins may prove instructive in elucidating the mechanism of protection afforded by pyridoxamine.

## 4 Discussion

Here we have shown that cognitive dysfunction occurred in rats with 8 weeks of chronic diabetes. Using the NOR test, we demonstrated that diabetic rats have disturbed recognition memory after an ITI of 1 h, but not 3 min, which indicated likely hippocampal dysfunction in diabetes. We showed that this deficit is ameliorated by treatment with insulin or pyridoxamine. We identified and quantified specific molecular changes in metabolites and proteins within the hippocampus using a comprehensive untargeted ‘omics approach and highlighted changes in the polyol metabolic pathway and proteins associated with glutamatergic signaling and oxidative stress in diabetes. The effect of pyridoxamine on metabolic profiles, cytoskeletal pathways and cell signaling pathways in the hippocampus was also described.

Control rats tend to spontaneously explore new objects and can discriminate between novel and familiar objects. This exploratory behavior has been used to assess deficits in recognition memory in multiple disease models [26], since it can be inferred that the rat formed a memory of previously exploring the familiar object during the acquisition phase. The NOR test was chosen as it is simple, ethologically-relevant, does not require training, rewards or food/water deprivation, and thus is particularly well-suited for diabetic rodents and supports the 3Rs and ARRIVE guidelines. One caveat is that blinding of control versus diabetic rats may not be entirely possible, due to the evident size difference between the groups. The ability of STZ-diabetic rats to distinguish the familiar from novel object after a 3 min ITI indicates that the localized cortical regions involved in the temporary storage of STM (e.g. prefrontal cortex), via transient modifications of pre-existing synaptic connections e.g. the alteration of neurotransmitter release, are not significantly impacted by diabetes. It also demonstrates that diabetic rats have the maintained visual acuity, curiosity to investigate, and ability to recognize the familiar object despite their disease state. It will be interesting however, to explore the use of additional assays of memory and behavior (e.g. spatial recognition memory in the Y maze) in future studies.

We found that insulin-treated diabetic rats have the ability to recognize the familiar object, therefore enabling us to ascertain that the cognitive deficit is a consequence of the diabetic phenotype (e.g. hyperglycemia, hyperlipidemia and/or hypoinsulinemia), rather than an off-target action of STZ. As well as its peripheral role in glucose utilization, insulin is pivotal to multiple central processes, including information processing essential for cognition. Impaired insulin signal transduction and tau hyperphosphorylation in whole brain homogenates have been associated with cognitive deficits in mice (9 weeks post-STZ [11]). During memory encoding and retrieval insulin-receptor signaling can modulate glutamatergic (via NMDA receptor potentiation) and GABAergic (via GABA receptor recruitment to the postsynaptic membrane) transmission, thereby regulating synaptic plasticity in the hippocampus [27].

To our knowledge, our study is the first unbiased and untargeted approach using GC–MS metabolomic profiling of the STZ-diabetic rat hippocampus, although a previous ^1^H NMR-based metabolomic analysis of 13 metabolites in different brain regions revealed the hippocampus to be susceptible to hyperglycemia associated damage with changes noted in metabolites of neurotransmitter synthesis and metabolism (choline, aspartate and lactate) [28]. The metabolomic profile of the hippocampus of diabetic rats resembles the pattern of abnormal glucose utilization observed in our previous characterization of PNS tissue (sciatic nerve, dorsal root ganglia, and trigeminal ganglia) of STZ-diabetic rats [22] and interestingly is also similar to studies of post-mortem brain samples from people with Alzheimer's disease [29] and Huntington's disease [30], potentially highlighting similar pathologies and an important risk factor for cognitive decline.

Pathway analysis of proteomic data revealed a significant up-regulation of pro-inflammatory pathway proteins (“*IL-12 signaling and production in macrophages*” and “*FXR/RXR activation*” of the acute phase response, as well as mechanisms of oxidative stress (“*Production of nitric oxide and reactive oxygen species in macrophages*” and “*Valine degradation*”) in the diabetic rat hippocampus. Activation and infiltration of macrophages is associated with ischemia and axonal degeneration in STZ-diabetic sciatic-tibial nerves [31]. It has also been shown that reducing inflammatory macrophages in the brains of STZ-diabetic rats inhibits the decrease in antioxidant defences, thereby suggesting that macrophages play a role in promoting oxidative stress [32]. The likely increased production of free radicals and the reduction in antioxidant defences in our rats could thereby hinder the brains ability to modulate damage leading to central dysfunction in diabetes.

There are similarities between the changes seen in STZ-diabetic rats and brain samples from people with Alzheimer's disease i.e. Aβ accumulation and associated neuroinflammation (via the activation of microglia and astrocytes which produce pro-inflammatory cytokines) causing neuronal injury and ultimately cognitive decline [8].

Our analyses highlighted glutamatergic disruption in the hippocampus of diabetic rats. The critical role of glutamate in learning and memory is well-established, particularly in the facilitation of synaptic transmission [33] and induction and maintenance of long-term potentiation in hippocampal neurons [34]. Glutamate degradation (to gamma-aminobutyrate (GABA)) may be markedly increased in the hippocampus of diabetic rats. Namely, levels of GAD1 and GAD2 (glutamate decarboxylase) were increased, a characteristic of “GABA shunt” activation (an alternative pathway of energy production typically activated during cellular stress [35]). Members of the solute carrier family membrane proteins of the “*Glutamate receptor signaling pathway*” including SLC17A6 and 7 (vesicular glutamate transporters [36]) and SLC1A2 and 4 (high affinity glutamate transporters [37]) were significantly down-regulated indicative of altered glutamate signaling and thus disruption of synaptic plasticity [38]. Similar alterations in glutamate signaling are reported in rodent models of Alzheimer's disease [39], [40] and schizophrenia (glutamatergic dysfunction in the cortex and hippocampus has been identified as an important mediator of impaired cognition [41]).

The biocatalytically active form of pyridoxamine (pyridoxal-5-phosphate) is essential to many biochemical pathways [42]. Pyridoxamine may prevent object recognition deficits by either protecting against diabetes-associated damage or by enhancing memory consolidation processes. There are thus numerous potential mechanisms through which pyridoxamine treatment could correct early LTM recognition deficits, e.g. counteracting (1) glucose-related metabolic dysfunction; (2) aberrant insulin signaling; (3) anti-inflammatory actions; (4) synaptic modifications; (5) anti-oxidative, anti-glycation effects and/or suppression of lipid modification; and/or (6) dysregulation of neurotransmitter synthesis.

Pyridoxamine treatment did not impact on systemic blood glucose levels but did reduce the plasma hyperlipidemia observed in diabetic rats, in agreement with other studies [16], [43]. Metabolomic analysis did not show any correction of the altered sugar and lipid metabolism pathways by pyridoxamine, but pyrophosphate and 2-pyrrolidinone were significantly up-regulated. 2-pyrrolidinone is a cyclization product of GABA [44] and has been shown to facilitate synaptic transmission in rat hippocampal slices by amplification of PKC and nicotinic α7 acetylcholine receptor activity [45]. Since α7 receptors modulate hippocampal activity (through release of glutamate and GABA) and α7 agonists and positive allosteric modulators can restore cognitive performance in a number of preclinical models for Alzheimer's disease [46] and schizophrenia [47], it would be interesting to investigate the effects of α7 agonists in diabetic rats in future studies.

IPA analysis revealed alterations in numerous pathways including ‘*Epithelial adherens junction signalin*g’ which largely includes actin- and tubulin-associated cytoskeletal molecules and may indicate altered neuronal plasticity in pyridoxamine-treated rats. By manually examining proteomic datasets, we found 82 protein targets whose levels were significantly changed in both datasets. Hippocampal proteins down-regulated in diabetes and up-regulated by pyridoxamine, include beta-actin and synaptopodin. In the hippocampus, synaptopodin is expressed in the dendritic spine neck of principal cells, and is considered critical to dendritic spine plasticity [48], [49]. Dendritic spines remodel their structure to adapt to changes in synaptic activity during learning and memory processing. The morphological adaptation occurs secondary to SYNPO-induced rearrangements in the postsynaptic actin cytoskeleton [49], which, in part, involves the association of the actin cytoskeleton with smooth endoplasmic reticulum calcium stores. The subsequent release of calcium leads to SYNPO-dependent delivery of AMPA receptor GluRI into dendritic spines, thereby increasing dendritic spine branching and motility [50]. Interestingly, our recent data found significant down-regulation of synaptopodin in the brains of people with Alzheimer's disease [51]. A similar diabetes-associated decrease in synaptopodin in the kidneys has also been described in db/db mice and this was also normalized by pyridoxamine treatment [52].

In conclusion we have demonstrated in multiple studies that robust recognition memory deficits in diabetic rats are correlated with dysfunction in brain metabolism, and altered protein expression (especially in pathways associated with synaptic plasticity and neurotransmission) in the hippocampus. Interestingly we found that pyridoxamine treatment prevented these recognition memory deficits, potentially by modulating neurotransmitter regulation and synaptic modification, although further investigation is needed. Our accessible datasets provide a valuable resource for researchers to search for particular proteins of interest and also for future mechanistic studies to elucidate the mechanisms by which the cognitive deficits have occurred in diabetes.

## Data availability

The datasets generated and analyzed in the current study are available from the Mendeley Data repository [https://doi.org/10.17632/n6z95235zx.1 and https://doi.org/10.17632/72n3ds7hhg.1].

## Funding

This research was funded by a Medical Research Council Studentship (MR/K501311/1), University of Manchester funding and MRC MR/L011093/1 (to AWD). The study sponsor was not involved in the design of the study; the collection, analysis, and interpretation of data; writing the report; or the decision to submit the report for publication.

## Contribution statement

SK and NJG contributed to study design, data collection and analysis, discussion and drafted the manuscript. JCN, BG, and NJG designed and supervised the NOR studies. SMR, CCR, and BG helped score some of the NOR experiments (Study 1 and 2). PB, SJC, and SK performed the GC–MS analysis and data analysis. RDU and SK performed the iTRAQ analysis. AWD and AMP developed and performed the Bayesian analyses. LAHZ performed Volcano analysis. All authors contributed to discussion, reviewed, and contributed to the final manuscript. GJSC, NJG, and RDU contributed to study design and are the guarantors of this work and, as such, had full access to all the data in the study and take responsibility for the integrity of the data and the accuracy of the data analysis. Parts of this study were presented in abstract form at the NEURODIAB Meeting (Diabetic Neuropathy Study Group), Portugal (9–11/09/17).
